# Value of large scale expansion of tumor infiltrating lymphocytes in a compartmentalised gas-permeable bag: interests for adoptive immunotherapy

**DOI:** 10.1186/1479-5876-9-63

**Published:** 2011-05-16

**Authors:** Thomas Zuliani, Julien David, Sylvain Bercegeay, Marie-Christine Pandolfino, Isabelle Rodde-Astier, Amir Khammari, Cécile Coissac, Bruno Delorme, Soraya Saïagh, Brigitte Dréno

**Affiliations:** 1 Cell and Gene Therapy Unit (UTCG): CIC biotherapy INSERM 0503 Hôtel-Dieu University Hospital 44093 Nantes cedex 01 France; 2 Dermatological Oncology Department: CIC biotherapy INSERM 0503 Hôtel-Dieu University Hospital 44093 Nantes cedex 01, France; 3 Immunodermatology Laboratory: CIC biotherapy INSERM 0503 Hôtel-Dieu University Hospital 44093 Nantes cedex 01, France; 4 MacoPharma, 59200 Tourcoing, France

## Abstract

**Background:**

Adoptive cell therapy (ACT) has emerged as an effective treatment for patients with metastatic melanoma. However, there are several logistical and safety concerns associated with large-scale *ex vivo *expansion of tumour-specific T lymphocytes for widespread availability of ACT for cancer patients. To address these problems we developed a specific compartmentalised bag allowing efficient expansion of tumour-specific T lymphocytes in an easy handling, closed system.

**Methods:**

Starting from lymph nodes from eight melanoma patients, we performed a side-by-side comparison of Tumour-Infiltrating Lymphocytes (TIL) produced after expansion in the compartmentalised bag versus TIL produced using the standard process in plates. Proliferation yield, viability, phenotype and IFNγ secretion were comparatively studied.

**Results:**

We found no differences in proliferation yield and cell viability between both TIL production systems. Moreover, each of the cell products complied with our defined release criteria before being administered to the patient. The phenotype analysis indicated that the compartmentalised bag favours the expansion of CD8+ cells. Finally, we found that TIL stimulated in bags were enriched in reactive CD8+ T cells when co-cultured with the autologous melanoma cell line.

**Conclusions:**

The stimulation of TIL with feeder cells in the specifically designed compartmentalised bag can advantageously replace the conventional protocol using plates. In particular, the higher expansion rate of reactive CD8+ T cells could have a significant impact for ACT.

## Background

Adoptive cell therapy (ACT) has been successfully implemented as a modality for the treatment of cancers for almost twenty years. The applications of this therapy for cancer have included treatment of hematopoietic cancers through the targeting of viral antigens [1], renal cancer carcinogenesis [2] and metastatic melanoma [3-5], with good evidence of efficacy. Important progress has been achieved in the field of melanoma and in recent clinical trials, objective response rates of between 50% and 70% have been obtained when combined with immunodepletion [6]. More recently, in a phase II study it was shown that at the metastatic stage, 43% of the patients with stage III and IV melanoma experienced an objective clinical response after treatment with autologous melanA/Mart-1-specific T lymphocyte clones [7]. These encouraging results emphasize the need to further develop this cancer therapy strategy.

ACT is an immunotherapy technique in which autologous tumour-infiltrating lymphocytes are isolated from resected metastatic lesions, expanded in culture, usually along with vaccines or growth factors, and re-administered to patients. In this approach very large numbers of TIL need to be generated and administered. The common procedure for lymphocyte expansion starts in multi-well plates or in T-flasks. TIL are initially stimulated with recombinant human IL-2, irradiated allogenic peripheral blood mononuclear cells and sometimes B-EBV cells as feeder cells. Expansion is then completed by transferring cells to gas-permeable bags in the presence of recombinant human IL-2. Although this procedure has proved its efficacy in generating large numbers of viable activated TIL, it presents some limitations in the context of GMP, especially during the expansion phase in multi-well plates/flasks. The main limitation is that during this period, cells need to be fed every 3-4 days and plates and flasks constitute an "open system", allowing potential contamination of the cell therapy product during handling. In addition, the large quantities of cells needed for each infusion require the use of multiple containers, which has two drawbacks; first, it can introduce variability in cell preparation and second, the handling procedures are labour-intensive and time-consuming. In order to make this stage of TIL production more standardised, safer and easier, we developed a specifically dedicated bag prototype for TIL expansion on feeder cells. The main aim was to facilitate cell-to-cell contact between TIL and feeder cells. This was achieved by dividing the bags into two asymmetric compartments: one small compartment into which TIL and feeder cells were injected, above a larger compartment containing the medium, separated by a discontinuous welding.

By comparative analysis of TIL produced using a standard multi-well plate method and in the specifically developed bags, we report here that bags can advantageously replace plates. First, bags have the advantage of being a safe, closed system which is much easier to handle than plates. Second, TIL produced in bags were comparable to those produced in plates in terms of quantity and viability. Third, we found that the proportion of CD8+ T cells at the end of the production process was higher in bags. Finally, this higher proportion of CD8+ cells produced correlated with a higher number of CD8+ T cells producing IFNγ when TIL were placed in contact with the autologous melanoma cell line.

## Methods

### Tumour samples and cell line

Tumour samples were obtained from 8 patients with melanoma-invaded lymph nodes (LN). All patients signed an informed consent form approved by the Ethics Committee (Pays de La Loire) for the use of surgical samples for research. All samples were immediately transferred to the cell and gene therapy unit following surgical resection. LAZ 388 cell line, an Epstein Barr virus-transformed B-cell line, was kindly provided by Thierry Hercend.

### Establishment of autologous melanoma cell lines

Melanoma cell lines were established as previously described [8]. Briefly, fresh lymph nodes with metastasis were minced into small tumour explants (approximately 1-2 mm^3^) with scissors and a biopsy punch. The resulting fragment suspension was centrifuged, and then pieces were inoculated (at a rate of 2 or 3 per well) into the wells of a 24-well plate (NUNC) and 1.5 ml per well of RPMI/FCS (10%) was added. The plates were placed at 37°C in a humidified incubator with 5% CO_2_. The plates were observed under a light microscope every week and sub-cultured if necessary.

### TIL generation

**Figure 1:**
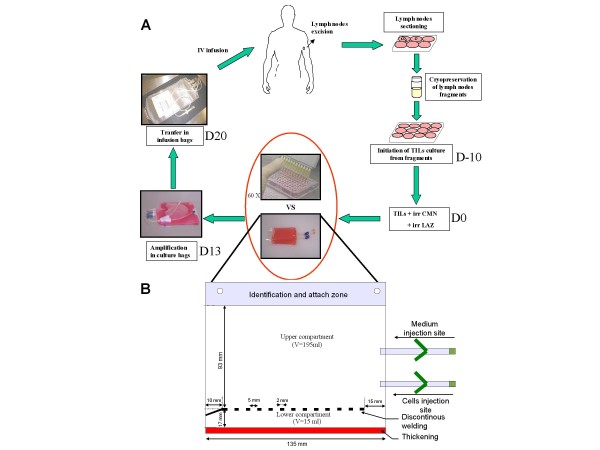
**TIL amplification after stimulation in multiple 96-wells plates or in bags.** A, Illustration of the TIL production process for ACT. Briefly, lymph nodes are excised from patients and sectioned for cryopreservation. If the patient is included in the protocol, node fragments are thawed and TIL cultured in 12-well plates for 10 to 14 days. Then, TIL together with feeder cells (irradiated PBMC and irradiated LAZ cells) are plated in sixty 96-well plates for stimulation. Ten days later, TIL are pooled and expanded in culture bags before being administered to patients (see "Materials and Methods" for details). Even though 96-well plates have the advantage of enhancing TIL stimulation through close contact between TIL and feeders cells, they represents a time- and labour-intensive step in the TIL production process. Furthermore, as it requires multiple containers (i.e. sixty 96-well plates), this method is a source of potential contamination and variability of the cell therapy product. In order to make TIL stimulation with feeder cells safer and more straightforward, we developed a specific compartmentalised bag. B, Schematic representation of the compartmentalized bag. This bag is composed of a size-reduced lower compartment (L, 135 mm × H, 17 mm; Vmax 15 ml) that allows close contact between cells. This compartment has been thermoformed to avoid plastic sticking and facilitate cell injection. TIL and feeder cells are injected into this compartment through the bottom injection site. Then culture medium is added in the larger upper compartment (L, 135 mm × H, 93 mm; Vmax 195 ml) via the upper injection site. The two compartments are separated by a discontinuous welding (forteen 2 mm weldings separated by 5 mm) that allows exchanges between both compartments but prevents the passage of cells from the lower to the upper compartment during culture medium renewal.

TIL were produced according to a procedure described previously [9] (See Figure 1 for an illustration of the TIL production process for ACT). Briefly, TIL were isolated from 8 tumour samples by culturing cryopreserved fragments of stage III tumour-invaded lymph nodes in two 12-well tissue culture plates with X-vivo 15 serum-free medium (Lonza) containing 150 U/ml IL-2 (Novartis), for 10 to 14 days. To perform large-scale expansion, 0.39 × 10^6 ^TIL from these short-term culture were plated in thirteen U bottom 96-well microplates at a density of 300 viable lymphocytes/well with irradiated feeder cells in 150 μl of IL-2 medium. For 0.39 × 10^6 ^TIL, 26 × 10^6 ^irradiated LAZ cells and 52 × 10^6 ^allogenic PBMC were used as feeder cells. PHA-L (Sigma-Aldrich) was added on day 0 (15 μg/ml). After three days, most of the remaining PHA-L was removed by replacing the culture medium. Cells were cultured for a further 10 days and fed every 3-4 days by removing 1/3 of the medium and by replacing it with fresh X-vivo15 + 150 UI/ml IL-2. This is the protocol that is currently run for a clinical phase III study (NCT00200577), except that in order to obtain sufficient cells for patient infusion (>10^9 ^cells), 1.8 × 10^6 ^cells are plated with the feeder cells, the equivalent of plating sixty 96-well plates. In this study, we compare in parallel the stimulation/expansion of 0.39 × 10^6 ^TIL grown with feeder cells in thirteen 96-well plates versus the same quantity of TIL grown with the same quantity of feeder cells in one specially manufactured bag (MacoPharma, french patent 07/00252). This polyolefin bag consists of a small compartment (bottom part) and a larger one (upper part), separated by a discontinuous welding allowing medium exchange between both compartments. Firstly, TIL and feeder cells are injected into the bottom compartment of the bag, diluted in 10 ml X-vivo 15 medium containing 150 UI/ml IL-2. Then, 185 ml medium containing PHA-L are carefully added to the upper compartment of the bag to avoid cell transfer from bottom to top. For both the plate and bag conditions, thirteen days after plating with feeder cells TIL were recovered, adjusted to 1 × 10^6 ^cells/ml in 150 UI/ml IL-2 X-vivo 15 medium and transferred into standard Lifecell culture bags (Baxter fenwall) for expansion for a further 7 days (Figure 1). It has to be noted that the Baxter expansion bags whose production has recently been stopped could efficiently be replaced by uncompartmentalized Miltenyi bags or Mabio Clinicell system. Three days after transfer to the expansion bags, cells were fed with fresh X-vivo15 medium containing 150 UI/ml IL-2, readjusted to 1 × 10^6 ^cells/ml and grown until day 20. TIL in bags and in plates were then recovered at day 20 for immunophenotypic characterization and to assess IFNγ production by TIL in the presence of the autologous melanoma cell line. Immunophenotypic characterization was also performed at days 13 and 17 of the expansion process.

### Flow cytometry analysis of CD3, CD4, CD8 and CD19 cell populations

At several stages of TIL generation (days 13, 17 and 20), we performed flow cytometry analysis to monitor CD3, CD4, CD8 and CD19 expression. Briefly, 0.2 × 10^6 ^cells were rinsed twice in PBS. Cells were then stained simultaneously with anti-CD3-PC5 mAb and either anti-CD4 FITC mAb, anti-CD8 FITC mAb or anti-CD19 FITC mAb for 30 minutes at 4°C protected from light. Isotype matched controls were performed under the same conditions. All the Abs were purchased from BD Bioscience (France) except the anti-CD3-PC5 mAb, which was purchased from Beckman/Coulter (Marseille, France). Finally, cells were rinsed twice in PBS and resuspended in PBS/PFA (1%) until the flow cytometry analysis. A minimum of 10^4 ^viable cell gated events were analysed on a FACScalibur flow cytometer using Cell Quest software (Becton Dickinson, Grenoble, France). Data were reanalysed with winMDI software (developed by JC Trotter).

### IFNγ production by TIL in response to autologous melanoma cell lines

Approximately 10^5 ^lymphocytes were stimulated by 3 × 10^5 ^autologous melanoma cells in 200 μL of X-vivo 15 medium in the presence of brefeldin A, 10 μg/ml (Sigma, St Louis MO, USA) in round-bottom 96-well plates. The culture was incubated for 6 h at 37°C in a 5% CO_2 _humidified atmosphere. Cells were stained for surface markers with fluorochrome-labelled monoclonal antibodies (anti-human CD4-APC, anti-human CD8-FITC, BD Biosciences, France). For intracytoplasmic IFNγ staining, cells were fixed for 10 min at room temperature in a 4% paraformaldehyde solution in PBS (Sigma), then washed and stored at 4°C until labelling. Fixed stimulated lymphocytes were stained for IFNγ production according to the previously described method [10]. Anti-IFN-γ specific antibody was purchased from BD Biosciences, France. After staining, cells were resuspended in PBS until the flow cytometry analysis. A minimum of 10^4 ^cells were analysed on a FACScalibur flow cytometer using Cell Quest software (Becton Dickinson, Grenoble, France). Data were reanalysed with winMDI software.

### Statistical analyses

Results are expressed as mean ± SEM. The statistical differences between values were determined by means of the Wilcoxon matched pairs test. A difference between values was considered statistically significant if p-value < 0.05.

## Results

### Cell recovery and viability are similar when TIL are initially co-cultured with feeder cells in plates or in bags

**Figure 2:**
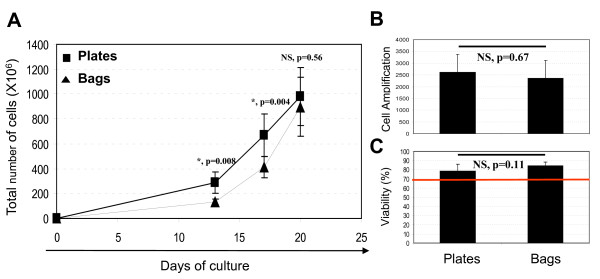
**Comparison of TIL proliferation in plates and bags**. **A**, Total quantity of TIL produced in plates and bags during the culture period. **B**, Cell amplification of TIL produced in bags and plates between day 0 and day 20. **C**, Viability of TIL produced in bags and in plates at the end of the process (day 20). The data are the means of experiments carried out on triplicate from 8 donors. p-values are shown.

Currently, TIL produced for clinical use should comply with specific set criteria which characterize the final product before it is administered to the patient. Among them, cell viability must be ≥70% and the infusion dose higher than 10^9 ^viable TIL. This dose corresponds to a cell production initiated with 1.8 × 10^6 ^TIL obtained from lymph node fragments and co-cultured with feeder cells in sixty 96-well plates. At the end of the process, it corresponds to a 555-fold amplification. The prototype of the bag developed to replace the expansion phase in plates could be loaded with a maximum of 0.39 × 10^6 ^TIL, corresponding to an equivalent of thirteen 96-well plates (see materials and methods). Starting with 0.39 × 10^6 ^TIL in one bag compared to 13 plates, we obtained a similar quantity of cells at the end of the process. Hence, at day 20 we recovered a mean of 1021 × 10^6 ^± 296 cells produced in plates versus 922 × 10^6 ^± 282 produced in bags for all the donors (Figure 2A). Extrapolated to standard production conditions (starting from 1.8 × 10^6 ^TIL), this corresponded to a yield of approximately 4706 × 10^6 ^cells in plates versus 4250 × 10^6 ^cells in bags and a 2618-fold and 2361-fold expansion respectively (Figure 2B). As regards cell viability, it was always over 70% for all the donors, with a mean of 84.9% ± 4.3 in bags versus 79.6% ± 7.2 in plates at day 20 (Figure 2C).

### Selective expansion of CD8+ cells in bags

**Figure 3:**
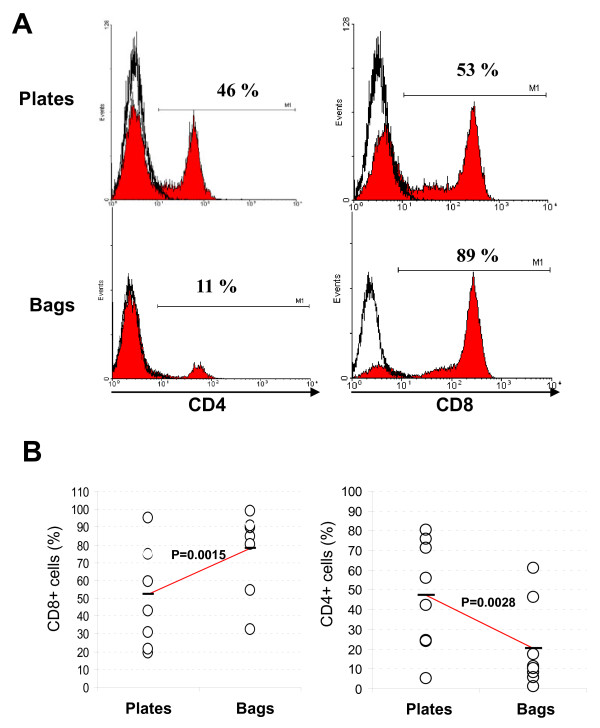
**CD4 and CD8 expression by TIL produced in plates vs bags**. **A**, representative cytometry histograms of TIL produced in plates (upper panels) and bags (lower panels) stained for CD4 (left panels) and CD8 (right panels) from patient 06. White histograms correspond to isotypic controls. **B**, graphical representation of the percentage of CD8 (left) and CD4 (right) positive cells produced in plates vs bags. Circles correspond to the mean of the percentage of CD8 and CD4 positive cells obtained for each donors. For both graphics, left and right circles correspond to TIL expanded in plates and bags respectively. For each line the horizontal bar corresponds to the mean of the 8 donors. The difference between means is indicated by the p-value.

We examined the phenotype of cells produced in both bag and plate test containers. CD3, CD4, CD8 and CD19 expression were analysed at day 13 (end of feeder cell stimulation), at day 17 and at the end of the expansion period at day 20. For all the samples, the percentage of CD3+ cells was always ≥98%. This high percentage of CD3+ cells was confirmed by the fact that fewer than 1% of CD19+ cells were found during cell expansion for all the samples (data not shown), confirming that LAZ cells were no longer proliferating after irradiation. CD3+ cells were further examined by double staining with either CD4 or CD8. The results for CD4 and CD8 expression of cells produced in plates and in bags are presented in Table 1 (day 20). Percentages of CD4 and CD8 at day 13 and day 17 were not significantly different than at day 20 (data not shown). Figure 3A illustrates the phenotypes of recovered TIL at day 20 for the patient 06 after expansion in plates versus in bags. Figure 3B illustrates the percentages of CD4+ and CD8+ cells produced in plates and in bags for each donor. For all the donors, the mean percentage of CD8+ cells in bags is 78% ± 23 versus 52.4% ± 28 in plates and the mean percentage of CD4+ cells is 20.1 ± 21.6 in bags versus 47.2 ± 28 in plates.

**Table 1:** Proliferation yield and phenotype analysis of lymphocytes expanded from metastatic lymph nodes of eight patients with melanoma

Patients	Initial cell number	Final cell number	Fold expansion	**CD4 **^**a**^		**CD8 **^**a**^		CD8+/IFNγ+ TIL	
				
	**(×10**^**6**^**)**	**(×10**^**6**^**)**		%	**Nb (10**^**6**^**)**	%	**Nb (10**^**6**^**)**	%	**Nb (10**^**6**^**)**
01 (plates)	0.39	794 (±501)	2035 (±1287)	56 (±8)	439 (±257)	43 (±9)	342 (±257)	2 (±1)	5 (±2)
01 (bag)	0.39	1128 (±445)	2892 (±1141)	11 (±2)	123 (±35)	85 (±2)	953 (±366)	14 (±1)	129 (±45)

02 (plates)	0.39	982 (±50)	2518 (±128)	80	797 (±49)	19 (±4)	191 (±54)	2 (±1)	3 (±1)
02 (bag)	0.39	1215 (±153)	3115 (±393)	17 (±6)	207 (±50)	80 (±3)	981 (±166)	5 (±1)	45 (±14)

03 (plates)	0.39	1698 (±182)	4352 (±466)	5 (±1)	85 (±19)	95 (±1)	1612 (±172)	15 (±8)	241 (±106)
03 (bag)	0.39	773 (±366)	1984 (±938)	1 (±0)	6 (±4)	99 (±0)	766 (±362)	15 (±5)	107 (±31)

04 (plates)	0.39	895 (±217)	2296 (±557)	76 (±2)	676 (±158)	21 (±2)	192 (±55)	1 (±1)	2 (±0)
04 (bag)	0.39	600 (±240)	1539 (±615)	61 (±18)	387 (±257)	33 (±16)	180 (±94)	2 (±1)	3 (±2)

05 (plates)	0.39	989 (±180)	2537 (±463)	24 (±4)	236 (±66)	75 (±4)	741 (±132)	6 (±1)	46 (±9)
05 (bag)	0.39	969 (±88)	2484 (±225)	8 (±3)	76 (±17)	90 (±2)	870 (±100)	12 (±4)	107 (±44)

06 (plates)	0.39	910 (±199)	2334 (±509)	42 (±4)	387 (±119)	60 (±7)	533 (±60)	5 (±1)	26 (±3)
06 (bag)	0.39	1323 (±152)	3393 (±389)	10 (±5)	132 (±71)	91 (±5)	1205 (±162)	23 (±2)	281 (±58)

07 (plates)	0.39	1126 (±199)	2889 (±509)	24 (±5)	274 (±73)	75 (±2)	851 (±174)	16 (±4)	132 (±27)
07 (bag) (plates))	0.39	795 (±174)	2039 (±446)	6 (±1)	46 (±18)	93 (±1)	737 (±162)	26 (±8)	184 (±29)

08 (plates)	0.39	772 (±123)	1982 (±316)	71 (±12)	552 (±142)	31 (±13)	234 (±81)	2 (±1)	4 (±1)
08 (bag)	0.39	571 (±163)	1465 (±417)	46 (±17)	281 (±182)	54 (±14)	296 (±24)	5 (±3)	14 (±9)

For each patients, 3 or 4 independent cell expansions were conducted in parallel in bags and in plates and analysed at day 20. Values are the means obtained from these independent cell amplifications. Bracketed values show the standard deviation.

^*a*^*, gated on lived CD3 + cells*.

### In response to autologous melanoma cell line stimulation, TIL in bags strongly express IFNγ compared to TIL in plates

**Figure 4:**
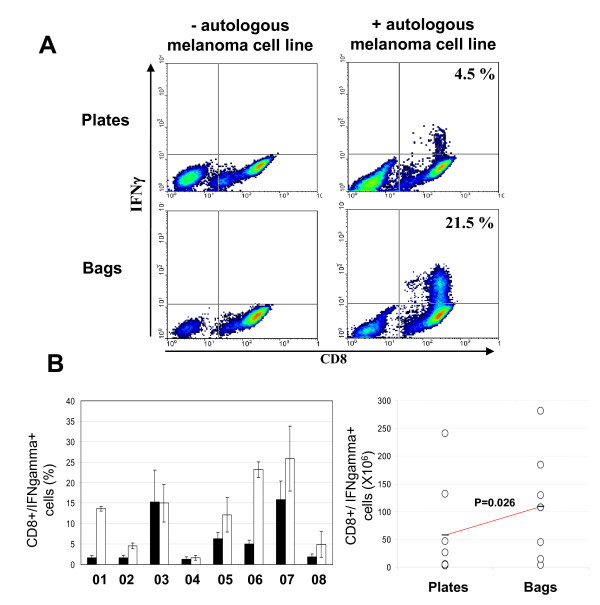
**IFNγ expression by TIL produced in plates vs bags**. **A**, representative cytometry dot plots of cells produced in plates (upper panel) and bags (lower panel) double stained for CD8 and IFNγ (see "Materials and Methods") after 6 hours in contact (right panel) or not (left panel) with the autologous melanoma cell line. Representative data obtained from patient 06. Percentages of CD8+/IFNγ+ cells are shown. **B**, graphical representation of the percentage of CD8+ cells expressing IFNγ in plates (black histograms) and in bags (white histograms) for all donors. **C**, Total number of CD8+/IFNγ expressing cells produced in plates versus bags. Circles correspond to the mean of CD8+/IFNγ expressing cells obtained for each donors. Left and right circle lines correspond to TIL expanded in plates and bags, respectively. For each line the horizontal bar corresponds to the mean of the 8 donors. The difference between these means is indicated by the p-value.

Following 20 days of expansion, TIL grown after feeder cell stimulation in plates and in bags were tested for their ability to produce IFNγ in response to autologous melanoma cell line stimulation. Figure 4A illustrates the density plots of CD8 and IFNγ expression following stimulation by the autologous melanoma cell line of TIL expanded in plates and in bags from the patient 06. No IFNγ production was seen when TIL were cultured in the absence of the autologous melanoma cell line. Six out of eight donors (donors 01, 02, 05, 06, 07 and 08), showed a higher percentage of specific TIL expressing IFNγ when cells were stimulated in bags compared to stimulation in plates. For the other two donors (03 and 04), these percentages were equal (see Table 1 and Figure 4B). For all the patients, the mean percentage of CD8+/IFNγ+ TIL was 12.5 ± 8.7 in bags versus 6.12 ± 6.1 in plates. Since the percentage of CD8+ cells is higher when cells are stimulated in bags, it results in a higher quantity of total CD8+/IFNγ+ cells produced in bags versus standard plates, 109 × 10^6 ^± 93 and 57 × 10^6 ^± 86 respectively (Figure 4C).

## Discussion

We have undertaken the improvement of the *ex vivo *TIL expansion process by initiating the stimulation/expansion phase with feeder cells in a specifically designed gas-permeable compartmentalized bags. We demonstrated that these bags can efficiently replace the standard protocol using plates for TIL stimulation/expansion in term of safety, yield and specificity of the TIL produced.

The potential antineoplastic efficacy of ACT is now well established and recent clinical trials have demonstrated the possibility of inducing tumor regression in patients with melanoma. However, ACT has some limitations for the widespread development of this therapeutic approach due to cost and safety concerns related to *ex vivo *cell amplification and any improvement of existing protocols for TIL expansion would participate to the development of availability of ACT. Recently some bioreactors have proven useful for some clinical applications with lymphocytes. More specifically, a bioreactor developed at Aastrom Biosciences has been tested to replace the second step of TIL expansion process in T-flasks/bags with OKT3, feeder cells and high concentration IL-2. This system has been reported to be suitable to expand TIL by safety and efficacy assays [11]. However, its use was not recommended by the authors for TIL infusion in ACT clinical trials to treat patients with advanced melanoma; the major negative aspects was the reduced cell yield compared to the current protocols and also the fixed and non-scalable design of the system that limits opportunity for in-process monitoring of the cell product and impedes cell concentration adjustments during the amplification.

Currently, TIL expansion relies on a first "open" step which consists of a micro culture initiated from tumor fragments or a single-cell digestion that are performed on multiwell plates. During this phase, TIL can be selected for their reactivity against autologous melanoma cell line before expansion. The second step, which consists in the rapid amplififcation of TIL obtained from tumor, is also initiated in an "open" system. In our group, TIL are first stimulated and expanded in multiple 96 well plates with low IL-2 concentration, PHA-L, with pooled CMN and LAZ as feeder cells before being transferred in gas permeable bags (see material and methods). In an other routinely used protocol, TIL are expanded first in multiple T-175 flasks with pooled CMN as feeder cells in presence of OKT3 and high IL-2 concentration. Although, the use of multiple 96-well plates has the disadvantage of being labour intensive compared to T-175 flasks, we observed that T-175 flasks were not suitable for optimal expansion of TIL with our amplification system using PHA-L, LAZ and low IL-2 concentration. Nevertheless, the use of either multiple T-175 flasks or 96-well plates represent another "open" step in the TIL amplification process. Thus, in order to both secure and also facilitate stimulation and expansion of TIL in an easy handling reproducible system, we developed a specific vessel.

Chemical and physical properties of containers in which cells are grown, could have significant impact on cell proliferation as well as differentiation. In the first step of development of an optimised device for TIL amplification on feeder cells, we designed two similar uncompartmentalized bags, one in EVA (ethyl vinyl acetate) and one in polyolefin, both materials being commonly used for cell culture bags. Results indicated that polyolefin bags allowed greater TIL amplification than EVA ones. However, when they were grown in uncompartmentalized polyolefin bags, TIL represented only 40% of the amount of TIL obtained according to our standard process in 96-well plates (data not shown). We finally designed another prototype in which a size-reduced compartment was created to locally increase cell concentration in the bag and enhance cell to cell contact between TIL and feeder cells.

In this work, we demonstrate that TIL can be efficiently expanded after stimulation with feeder cells in the specifically designed compartmentalised bag (Nantes university hospital patent 07/00238). In all cases, starting the production from eight lymph nodes from patients with advanced melanoma, we were able with this bag to produce the same quantity of cells at the end of the TIL expansion process as with our conventional method with plates. Moreover, proliferation yield and viability were compatible with the release criteria for patient infusion stated in our current TIL clinical protocol. Interestingly, we also showed that the ratio of CD8+/CD4+ cells is different between TIL produced in bags or in plates. Bags favour the proliferation of CD8+ cells as compared to plates. In our study, the mean percentage of CD8+ cells from the 8 patients was 78%, versus 52.4% in plates. The reason why the compartmentalised bag repeatedly induces more CD8+ TIL proliferation than plates remains at the moment unknown. However, we can hypothesized that it is, a least in part, related to the polyolefin material component of the bag. Indeed, when TIL were first amplified in the uncompartmentalized polyolefin bag, we already observed that the percentage of CD8+ cells in bag was higher than in plates. Moreover, the fact that it was not observed with the similar uncompartmentalised EVA bag, argues for a role of the polyolefin in this phenomenon suggesting a crucial role of the container. By studying the reactivity of TIL to the autologous melanoma cell lines via IFNγ production, we also found that bags expand a significantly greater quantity of reactive CD8+ T lymphocytes than plates. These results may have a significant impact on the efficacy of adoptive cell therapy. Indeed, the anti-tumoral activity of CD8 T cells has been widely demonstrated in mice and humans [12,13]. The evidence indicates that the presence of infiltrated CD8 T cells within tumours is positively correlated with better prognosis in cutaneous melanoma [14] as well as in several other types of cancer [15,16]. Moreover, in our group, a direct correlation was demonstrated between the clinical outcome of patients with one invaded lymph node treated with TIL and the presence of CD8+ cells producing IFNγ that were injected into the patients [17].

It has been proposed that the extensive *in vitro *stimulation and expansion required to obtain high quantities of cells may impair the activity and proliferative potential of these cells *in vivo *once adoptively transferred [18,19]. The i*n vitro *expansion of T cells for ACT has been shown to induce progressive CD8+ T cell differentiation into a late effector state that makes T cells less effective in mediating anti-tumour responses *in vivo*. Hence, it is proposed that culturing tumour-infiltrating lymphocytes for a limited period of time *in vitro *would increase the lymphocyte population capable of mediating tumour regression *in vivo*. This was recently confirmed by the *in vitro *and *in vivo *demonstrations that minimally cultured TIL display optimal characteristics for ACT [20-22]. In this study, in order to perform a side-by-side analysis of TIL proliferation, cells were injected into the lower compartment of the bag with identical TIL and feeder cell ratios and total cell concentration to those used in the standard protocol with plates. Preliminary data indicate that by increasing the cell concentration in the lower compartment of the bag and reducing the feeder cell stimulation period, it is possible to increase cell recovery (data not shown). In addition, for technical and logistical reasons, the TIL expansion process in plates is initiated with 1.8 × 10^6 ^TIL, while far more TIL are usually obtained from melanoma lymph node explant cultures. The initial limited numbers of TIL used for amplification is primarily due to the fact that 1.8 × 10^6 ^TIL correspond to the plating of sixty 96-well plates, which is time-consuming and labour-intensive even for highly qualified staff, particularly in a GMP environment. Because bags are easier to handle it should be possible to start TIL expansion with far more than 1.8 × 10^6 ^cells. Moreover, in order to further increase cell yield we are also developing a larger compartmentalized bag based on this prototype. This could result in a shortening of the cell production process, which would be beneficial for the patient in terms of cell therapy product availability but also efficacy.

This compartmentalised bag could be useful in other situations in addition to a TIL expansion protocol for ACT. Other immunotherapy strategies rely on the generation/selection of antigen-specific T reactive lymphocytes. These cells may be obtained by peptide immunomagnetic sorting [23], cloning [7], or generated after activation by tumour antigen- or apoptotic body-pulsed dendritic cells [24,25]. Whatever the method for selecting or generating antigen specific T cells, they need to undergo large-scale expansion, which is primarily performed by feeder cell stimulation. This emphasises the usefulness of this type of compartmentalised bag.

## Conclusions

We report here the processing of a specific compartmentalised bag into which TIL and feeder cells are inoculated in a restricted volume and that can accurately replace the standard system using multiple 96-well plates, thus allowing exogenous expansion of TIL in a quicker, more easy-handling and transposable, safer and cost-effective way. Moreover we show that in addition to producing the same quantity of cells as the standard procedure using plates, the bag allows an improved expansion of specific CD8+ cells regarding IFNγ production of TIL co-cultured with the autologous melanoma cell line. Taken together our data represent a major improvement of the exogenous TIL expansion process and may contribute to the development and availability of ACT for the treatment of patients with cancers that can be treated by immunotherapy.

## List of abbreviations

TIL: tumor infiltrated lymphocytes; ACT: adoptive cell therapy; IFNγ: Interferon gamma; EVA: ethyl vinyl acetate; IL-2: interleukin-2; PHA-L: phytoheamagglutinin-L; PBMC: peripheral blood mononuclear cells.

## Competing interests

The authors declare that they have no competing interests.

## Authors' contributions

TZ and JD carried out the cell culture, immunoassays and analysis and interpretation of the data. They drafted the manuscript. SB have made substantial contribution in the study design. MCP have produced the autologous melanoma cell lines and have been involved in IFNγ production assays. AK contributed to patient inclusion. IR-A, CC and B Delorme have developed, designed and produced the bags for cell culture. SS and B Dréno supervised and participated in the study design, result interpretation and in the writing. B Dréno participated in the recruitment and clinical follow-up of the patients. All authors read and approved the final manuscript.
